# Supplementary figures and images for: 15N Metabolic Labeling Quantification Workflow in Arabidopsis Using Protein Prospector
Source: Front Plant Sci. 2022 Feb 15;13:832562. doi: 10.3389/fpls.2022.832562 (PMC8885517; doi:10.3389/fpls.2022.832562)

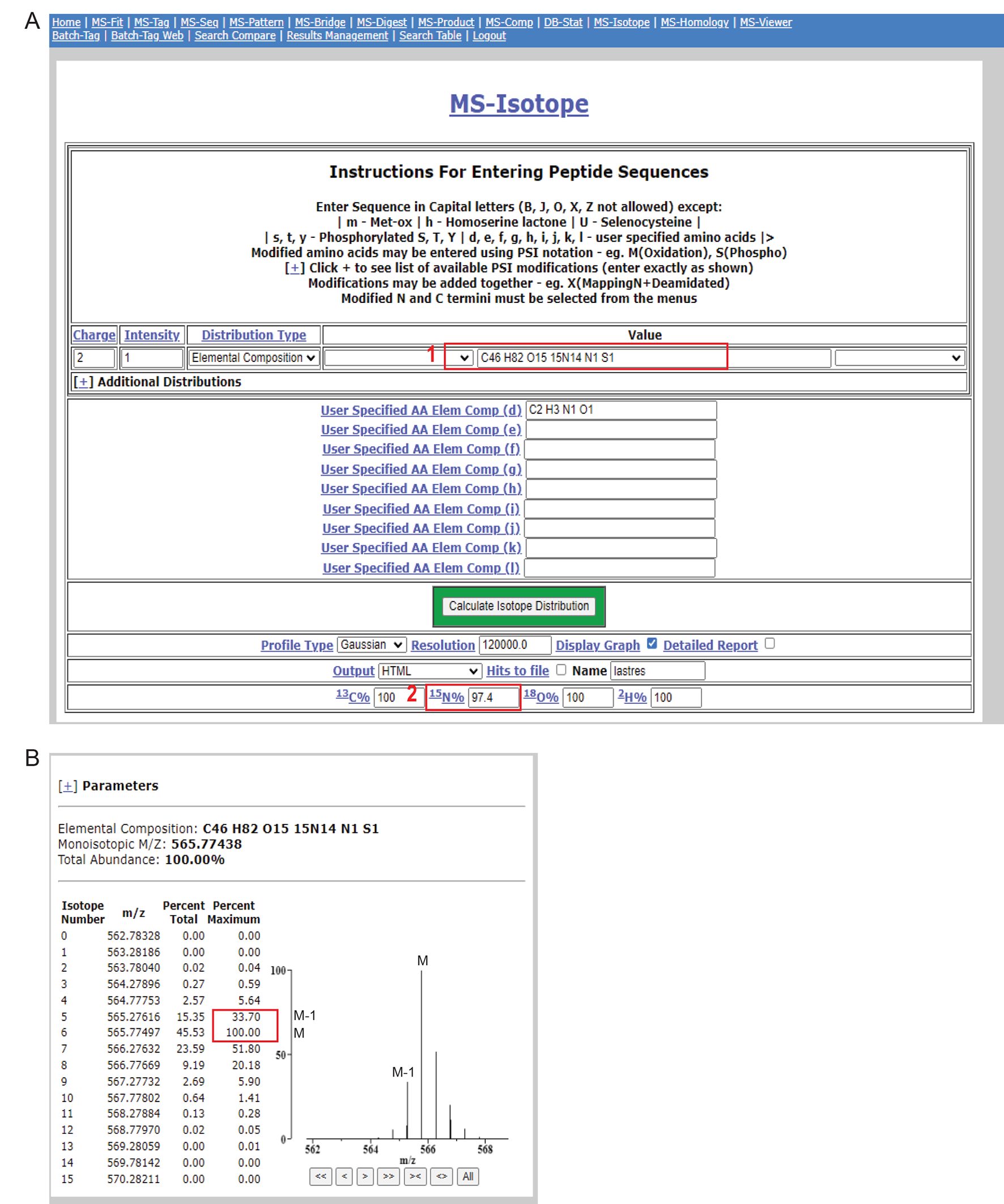

Supplement: Supplementary Figure 1 — Using MS-Isotope module in Protein Prospector to generate theoretical 15N peak pattern. (A) Users need to input either composition [1] or sequence of the peptide (not shown) and input the 15N incorporation rate [2]. (B) Protein Prospector will generate theoretical peaks and percentage number based on input from (A). [file Image_1.TIF]

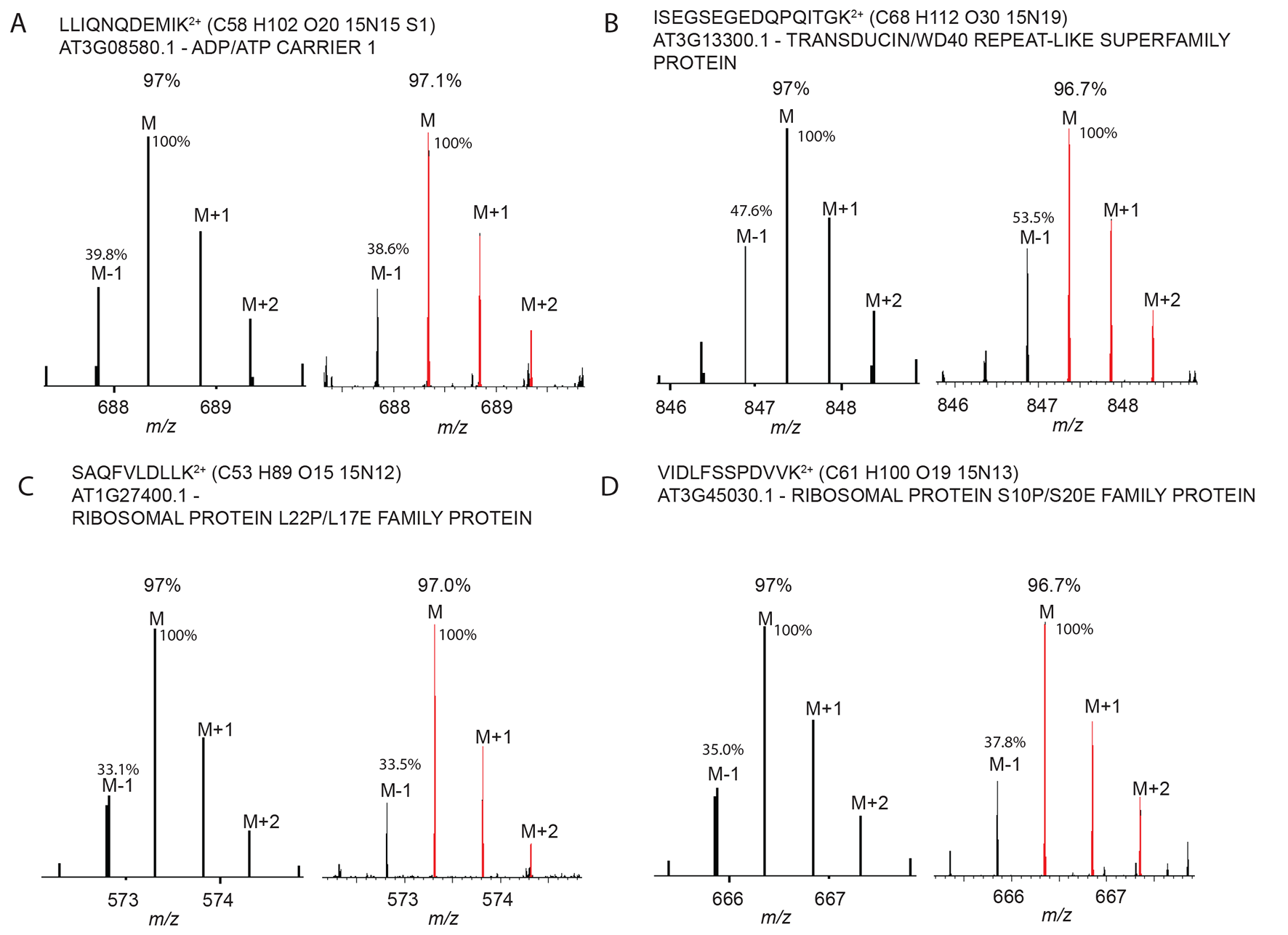

Supplement: Supplementary Figure 2 — Incorporation of the 15N into the Arabidopsis Col samples was 97%. (A–H) Eight 15N peptides from different proteins were examined to determine the labeling efficiency, with the left showing theoretical peak pattern at 97% labeling efficiency for all the peptides, and the right (M, M+1, M+2, colored in red) showing the experimental peak pattern and calculated labeling efficiency. Smaller m/z peptides are more preferred to determine the labeling efficiency, because the monoisotopic peak (M) is the largest peak and can be set as 100% maximum during calculation. [file Image_2.TIF]

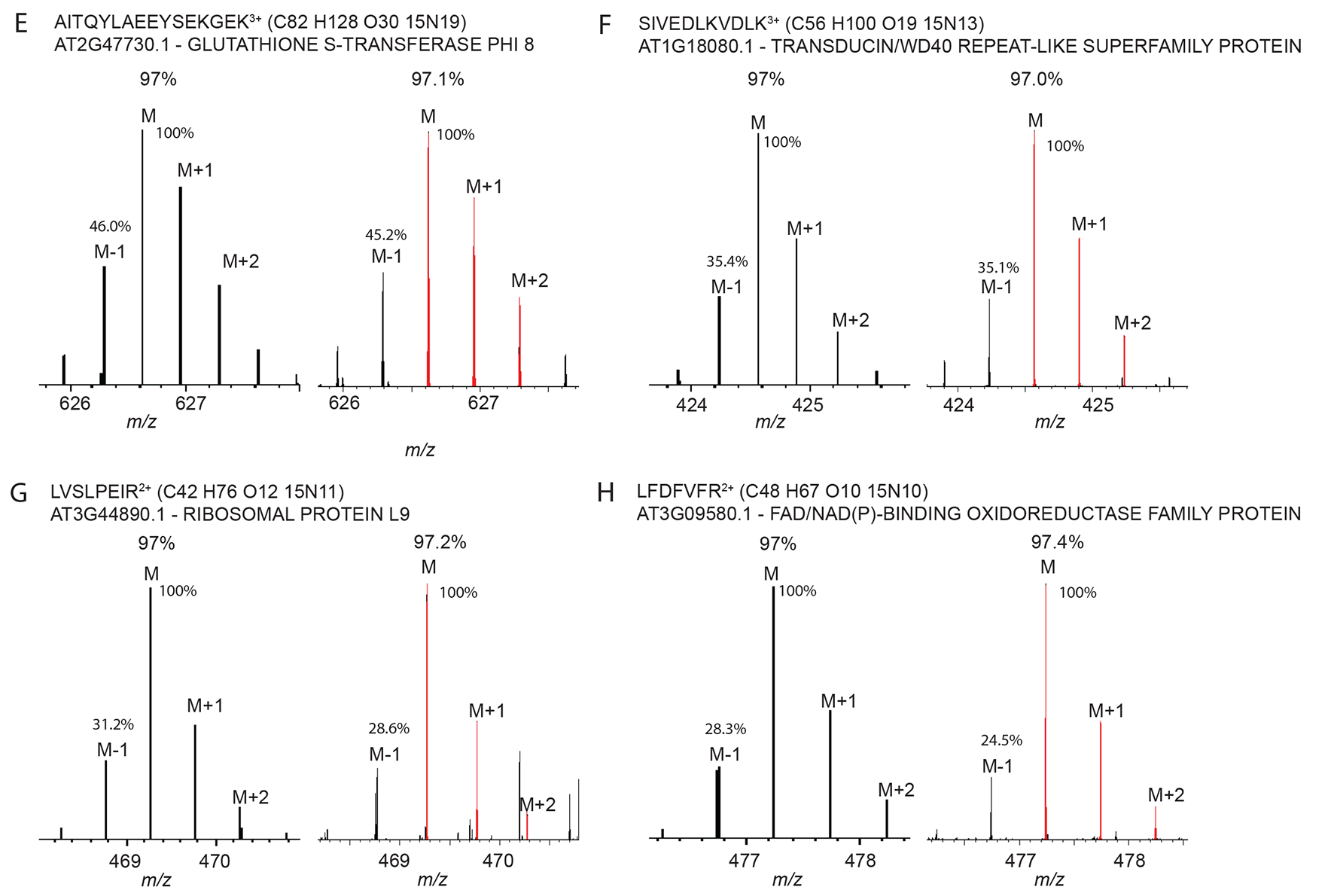

Supplement: Supplementary file 4 [file Image_3.TIF]
