# Supplementary material for: 15N Metabolic Labeling Quantification Workflow in Arabidopsis Using Protein Prospector
Source: Front Plant Sci. 2022 Feb 15;13:832562. doi: 10.3389/fpls.2022.832562 (PMC8885517; doi:10.3389/fpls.2022.832562)
Supplement: Supplementary Data 1 — R script used to generate the median and Q1/Q3 for quantification. [file Data_Sheet_1.docx]

**Supplemental Data 1: R script used to generate the median and Q1/Q3 for quantification.**

# ----------------------------------------------------------

# R script for plotting quantification from Protein Prospector

# ----------------------------------------------------------

# Inputs:

# Location of CSV file that contains the peptide sequence and protein names.

# CSV file must contain protein name and L.H.Intensities.

#Libraries required

library(tidyverse)

library(cowplot)

library(ggsci)

#Reading in csv with Proteins and L.H.Intensity

df = read.delim(file.choose(new = FALSE), header=T, sep=",", as.is=T)

#importing L.H.Intensity column as numeric

df$L.H.Intensity <- as.numeric(as.character(df$L.H.Intensity))

#Setting levels to protein column, optional

df$Protein = factor(df$Protein, levels = c("TUB2","ACT2", "SR45","PR5","PR1"))

#plot settings

g <-

ggplot(df, aes(x = Protein , y = L.H.Intensity, color = Protein)) +

labs(x = NULL, y = expression(paste(italic("acinus-2 pinin-1")," / Col (L/H) Intensity"))) +

theme_cowplot() +

scale_color_lancet()+

scale_y_continuous(limits = c(0.01,100),

breaks = c(0.01,0.02,0.05,0.1,0.2,0.5,1,2,5,10,20,50,100),

labels = c("Low",0.02,0.05,0.1,0.2,0.5,1,2,5,10,20,50,"High"),

trans = "log2") + #Log2 transformation, before boxplot calculations

theme(legend.position = "none",

text = element_text(size=7),

axis.text = element_text(size = 7))

#boxplot parameters

p <- g +

geom_boxplot(outlier.shape = NA,lwd=0.1) +

geom_point(position=position_jitterdodge(

jitter.width = 1.85,

jitter.height = 0,

dodge.width = NULL,

seed = 223),

size = .5,

alpha=1)

#saving final plot

ggsave(plot = p, width = 8.5, height = 8.5, dpi = 300, units = "cm",filename = "plot.pdf")
